# Spo13 prevents premature cohesin cleavage during meiosis

**DOI:** 10.12688/wellcomeopenres.15066.2

**Published:** 2019-09-02

**Authors:** Stefan Galander, Rachael E. Barton, David A. Kelly, Adèle L. Marston

**Affiliations:** 1 Wellcome Centre for Cell Biology, School of Biological Sciences, University of Edinburgh, Edinburgh, EH9 3BF, UK

**Keywords:** Meiosis, cohesin, Spo13

## Abstract

**Background:** Meiosis produces gametes through two successive nuclear divisions, meiosis I and meiosis II. In contrast to mitosis and meiosis II, where sister chromatids are segregated, during meiosis I, homologous chromosomes are segregated. This requires the monopolar attachment of sister kinetochores and the loss of cohesion from chromosome arms, but not centromeres, during meiosis I. The establishment of both sister kinetochore mono-orientation and cohesion protection rely on the budding yeast meiosis I-specific Spo13 protein, the functional homolog of fission yeast Moa1 and mouse MEIKIN.

**Methods:** Here we investigate the effects of loss of *SPO13 *on cohesion during meiosis I using a live-cell imaging approach.

**Results:** Unlike wild type, cells lacking *SPO13 *fail to maintain the meiosis-specific cohesin subunit, Rec8, at centromeres and segregate sister chromatids to opposite poles during anaphase I. We show that the cohesin-destabilizing factor, Wpl1, is not primarily responsible for the loss of cohesion during meiosis I. Instead, premature loss of centromeric cohesin during anaphase I in *spo13* *Δ* cells relies on separase-dependent cohesin cleavage. Further, cohesin loss in *spo13* *Δ* anaphase I cells is blocked by forcibly tethering the regulatory subunit of protein phosphatase 2A, Rts1, to Rec8.

**Conclusions:** Our findings indicate that separase-dependent cleavage of phosphorylated Rec8 causes premature cohesin loss in *spo13* *Δ* cells.

## Introduction

Sexual reproduction relies on a cell division programme called meiosis. In humans, this is highly error-prone and may give rise to infertility, miscarriage or chromosomal abnormalities such as Down syndrome (reviewed by Hassold & Hunt, 2001). Meiosis consists of two consecutive divisions, where homologous chromosome segregation in meiosis I is followed by mitosis-like sister chromatid segregation in meiosis II. Homologue segregation requires a number of adaptations to the chromosome segregation machinery ( Marston & Amon, 2004), including recombination of homologues, mono-orientation of sister kinetochores and the protection of pericentromeric cohesin in meiosis I.

Cohesin is a multi-subunit protein complex made up of the core subunits Smc1, Smc3 and the kleisin α-Scc1 ( Losada *et al*., 1998; Michaelis *et al*., 1997) as well as the accessory subunits Scc3 ( Tóth *et al*., 1999) and Pds5 ( Hartman *et al*., 2000; Panizza *et al*., 2000). In mitosis, cohesin resists the spindle forces that pull sister chromatids towards opposite poles, likely by topologically linking sister chromatids ( Gruber *et al*., 2003; Haering *et al*., 2002). Upon successful bi-orientation, securin (Pds1 in yeast) is ubiquitinated and destroyed by the proteasome, freeing separase (Esp1), which proteolytically cleaves Scc1 and thereby allows chromosome segregation.

Meiotic cohesin contains an alternative kleisin called Rec8 ( Buonomo *et al*., 2000; Watanabe & Nurse, 1999). Rec8 supports a number of meiosis-specific functions of cohesin, particularly during recombination. Rec8 cleavage is dependent on its prior phosphorylation by casein kinase 1δ (Hrr25), Dbf4-dependent kinase (DDK) Cdc7 ( Katis *et al*., 2010) and, potentially, Polo kinase (Cdc5) ( Brar *et al*., 2006). However, it is currently unclear how these kinases contribute to cohesin removal, with the role of Cdc5 in cohesin cleavage coming under particular scrutiny ( Attner *et al*., 2013; Argüello-Miranda *et al*., 2017; Brar *et al*., 2006; Galander *et al*., 2019; Katis *et al*., 2010). Hrr25 and Cdc7 are both independently sufficient for cohesin removal at anaphase I, most likely by promoting its cleavage ( Katis *et al*., 2010). Conversely, there is mounting evidence that Cdc5 facilitates cleavage-independent cohesin loss upon prophase exit ( Challa *et al*., 2019; Yu & Koshland, 2005), although a contribution to cleavage has also been argued ( Attner *et al*., 2013; Brar *et al*., 2006). While cohesin phosphorylation occurs along the length of the chromosome, the pericentromeric adapter protein shugoshin (Sgo1) binds protein phosphatase 2A (PP2A) to dephosphorylate Rec8 in the pericentromere and prevent its cleavage ( Katis *et al*., 2004a; Kitajima *et al*., 2006; Kitajima *et al*., 2004; Lee *et al*., 2008; Marston *et al*., 2004; Riedel *et al*., 2006; Tang *et al*., 2006). In meiosis II, Rec8 becomes deprotected by the action of Hrr25, which is thought to initiate Sgo1 degradation and phosphorylate Rec8 for cleavage ( Argüello-Miranda *et al*., 2017; Jonak *et al*., 2017).

In mammalian and *Drosophila* mitosis, cohesin is also removed in two steps. First, during prophase, Wapl opens the cohesin ring at the Smc3-Scc1 interface to trigger separase- and cleavage-independent cohesin removal ( Buheitel & Stemmann, 2013; Sumara *et al*., 2000; Waizenegger *et al*., 2000; Warren *et al*., 2000). A subset of cohesin is resistant to Wapl due to its prior acetylation and association with sororin ( Lafont *et al*., 2010; Nishiyama *et al*., 2010; Rankin *et al*., 2005; Rolef Ben-Shahar *et al*., 2008; Schmitz *et al*., 2007; Unal *et al*., 2008). Notably, pericentromeric cohesin is shielded from Wapl during mammalian mitosis by Sgo1-PP2A, which associates with, and dephosphorylates, both cohesin and sororin to prevent cohesin ring opening ( Kitajima *et al*., 2006; Liu *et al*., 2013b; McGuinness *et al*., 2005; Yamada *et al*., 2017). Second, upon sister kinetochore bi-orientation, Sgo1 relocalises from the kinetochore to the pericentromeric chromatin, and separase-dependent cohesin cleavage triggers anaphase onset ( Liu *et al*., 2013a; Liu *et al*., 2013b). A similar Wapl/Rad61-dependent, cleavage-independent cohesin removal pathway has been suggested to occur in budding yeast meiosis. Although condensin, Cdc5 and DDK have been identified as regulators of this pathway ( Challa *et al*., 2016; Challa *et al*., 2019; Yu & Koshland, 2005), budding yeast lacks an obvious sororin homologue. Thus, the mechanisms of Wapl-mediated cohesin removal in meiosis I are different to those in mammalian and *Drosophila* mitosis.

While previous research has identified key mechanisms governing cohesin protection, a number of additional proteins have been implicated in this process, but their roles remain unclear. Amongst them is the meiosis I-specific Spo13 ( Wang *et al*., 1987). Cells without *SPO13* only undergo a single meiotic division and show a variety of meiotic defects, including failure to mono-orient sister kinetochores in meiosis I and inability to protect cohesin ( Katis *et al*., 2004b; Klapholz & Esposito, 1980; Lee *et al*., 2004; Shonn *et al*., 2002). Spo13 is thought to have functional orthologs in both fission yeast (Moa1) and mouse (MEIKIN) ( Kim *et al*., 2015). The unifying feature of these proteins is their interaction with Polo kinases, whose kinetochore recruitment by Spo13, Moa1 and MEIKIN has been proposed to enable mono-orientation and cohesin protection ( Galander *et al*., 2019; Kim *et al*., 2015; Matos *et al*., 2008; Miyazaki *et al*., 2017).

The exact role of Spo13 in cohesin protection is currently unclear. Interestingly, *SPO13* overexpression blocks cohesin cleavage during mitosis ( Lee *et al*., 2002; Shonn *et al*., 2002; Varela *et al*., 2010), suggesting that Spo13 may also influence cohesin cleavage in meiosis, but how it might do so remains unresolved. Although Spo13 was implicated in ensuring the proper pericentromeric localization of Sgo1 ( Kiburz *et al*., 2005), other studies have found no difference in chromosomally associated Sgo1 ( Galander *et al*., 2019; Lee *et al*., 2004). In fact, it has been suggested that *spo13Δ* cells might retain residual pericentromeric cohesion in meiosis I ( Katis *et al*., 2004b).

 Here, we take a live cell imaging approach to re-evaluate the importance of Spo13 for cohesin protection. We show that both cohesin and sister chromatid cohesion are lost upon anaphase I onset in *spo13Δ* cells. Furthermore, we confirm that cohesin removal results from separase-mediated cleavage rather than removal by the prophase pathway. We also provide evidence that PP2A is capable of preventing cohesin cleavage in *spo13Δ* cells.

## Results

### Pericentromeric cohesin is prematurely lost in *spo13Δ* cells

**Figure 1.**
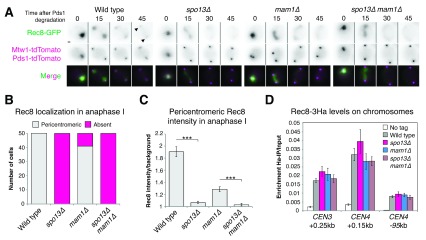
Cohesin is lost at anaphase I in the absence of *SPO13*. ( **A**) Representative images of Rec8-GFP, Mtw1-tdTomato and Pds1-tdTomato in live sporulating wild-type (AM13716), *spo13Δ* (AM15133), *mam1Δ* (AM15134) and *spo13Δ mam1Δ* (AM15135) cells. Scale bars represent 1 µm. Arrows indicate pericentromeric cohesin. ( **B**) The number of cells with pericentromeric Rec8-GFP in anaphase I is shown after scoring 50 cells from ( **A**). ( **C**) Rec8-GFP intensity was measured for 50 cells from ( **A**) in the area occupied by the tdTomato-labeled kinetochore protein Mtw1. ***p<0.001 (Welch two-sample t-test). ( **D**) Rec8 loading is unaffected by deletion of *SPO13*. Rec8-3Ha association with the indicated sites was measured in prophase I in wild-type (AM4015), *spo13Δ* (AM15343), *mam1Δ* (AM15342) and *spo13Δ mam1Δ* (AM15344) cells carrying *ndt80Δ* and a no tag control (AM11633). Cells were arrested in prophase by harvesting 5 h after resuspension in sporulation medium and anti-Ha ChIP-qPCR performed. Error bars show standard error of the mean from three independent biological experiments.

Previous analyses of fixed cells found that centromeric Rec8 is undetectable or greatly diminished in *spo13Δ* anaphase I cells ( Klein *et al*., 1999; Katis *et al*., 2004b; Lee *et al*., 2004). Further evidence that Spo13 is important for protection of centromeric cohesion came from the analysis of cells lacking the monopolin subunit, Mam1, which biorient, rather than monoorient sister kinetochores, yet fail to segregate sister chromatids due to the persistence of centromeric cohesion. Importantly inactivation of *SPO13* allowed *mam1Δ* cells to segregate sister chromatids during anaphase I ( Katis *et al*., 2004b; Lee *et al*., 2004). Together, these findings provide evidence that centromeric cohesion is impaired in *spo13Δ* cells. However, it has been argued that residual centromeric cohesin persists after securin destruction in *spo13Δ* cells and prevents timely spindle elongation ( Katis *et al*., 2004b). To clarify the importance of Spo13 in centromeric cohesion, we used live cell imaging of cells progressing through meiosis. We scored the percentage of cells where cohesin (Rec8-GFP) was retained at the pericentromere in anaphase I, as indicated by co-localization with the kinetochore protein Mtw1 ( Figure 1A, B). To ensure that observed effects in *spo13Δ* cells were not a consequence of mono-orientation loss, which partially impacts cohesion ( Nerusheva *et al*., 2014), we simultaneously imaged *mam1Δ* cells for comparison. Quantification of pericentromeric Rec8 ( Figure 1C) showed that, strikingly, deletion of *SPO13* leads to complete loss of cohesin in anaphase I. This is not due to impaired cohesin loading in early meiosis, since prophase I-arrested *spo13Δ* cells have similar levels of Rec8 on centromeres compared to wild type ( Figure 1D). We conclude that Spo13 is required for the retention of pericentromeric cohesin in anaphase I.

### *spo13Δ* cells prematurely segregate sister chromatids

**Figure 2.**
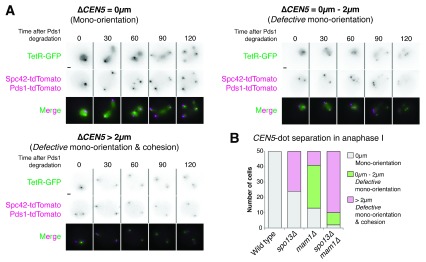
Deletion of *SPO13* permits sister chromosome segregation in anaphase I in *mam1Δ* mutants. ( **A**) Assay for mono-orientation and cohesion defects using heterozygous centromeric fluorescent markers. Representative images are shown. Scale bars represent 1 µm. Images for Δ *CEN5*=0µm, Δ *CEN5*=0-2µm and Δ *CEN5*>2µm were taken from wild-type, *mam1Δ* and *spo13Δ* cells, respectively. ( **B**) Frequency of *CEN5* distance categories is shown for the indicated genotypes after live-cell imaging. Wild-type (AM15190), *spo13Δ* (AM15118), *mam1Δ* (AM15119) and *spo13Δ mam1Δ* (AM15120) cells carrying *SPC42-tdTomato*, *PDS1-tdTomato* and heterozygous TetR-GFP foci at *CEN5*, were sporulated for 2.5 h before imaging on a microfluidics plate.

To assess sister chromatid cohesion in *spo13Δ* cells, we labelled one copy of chromosome V near the centromere with an array of tet operators ( *tetO*), expressed GFP-tagged TetR repressor ( Michaelis *et al*., 1997) and imaged *CEN5*-GFP foci in live meiotic cells. Upon anaphase I entry (as judged by degradation of yeast securin Pds1 ( Salah & Nasmyth, 2000)), three different phenotypes may be observed, depending on whether cells successfully mono-orient sister kinetochores and protect pericentromeric cohesin ( Figure 2A). In wild-type cells, a single GFP focus segregates to one of the spindle poles (as marked by the spindle pole body component Spc42-tdTomato). Alternatively, in case of defective mono-orientation, split GFP foci stay in close proximity (<2 µm) because sister chromatids are cohered by pericentromeric cohesin. Lastly, in cells lacking both mono-orientation and sister chromatid cohesion, GFP foci split over a greater distance (>2 µm). Note that, using this assay, pericentromeric cohesion loss during anaphase I can only be readily observed where it is accompanied by sister kinetochore bi-orientation. We subsequently scored the number of cells falling into either of these categories for each of the mutants analysed. This revealed that sister centromeres separate over large (>2 µm) distances in the half of *spo13Δ* anaphase I cells that bi-orient sister kinetochores ( Figure 2B), consistent with all cohesion being lost. A small fraction of centromeres in *spo13Δ mam1Δ* cells, which bi-orient almost exclusively, stay in close proximity in the 30-minute time frame measured ( Figure 2B), indicating that these cells at least temporarily retain sister chromatid cohesion. However, the loss of cohesion in all *spo13Δ* cells with bi-oriented kinetochores, the near-complete absence of Rec8, and the fact that deletion of *SPO13* permits efficient sister chromatid segregation in most *mam1Δ* cells ( Figure 2B) ( Katis *et al*., 2004b; Lee *et al*., 2004) together confirm that pericentromeric cohesion is predominantly non-functional in *spo13Δ* anaphase I cells.

### Restoring the second nuclear division in *spo13Δ* cells does not prevent chromosome missegregation

**Figure 3.**
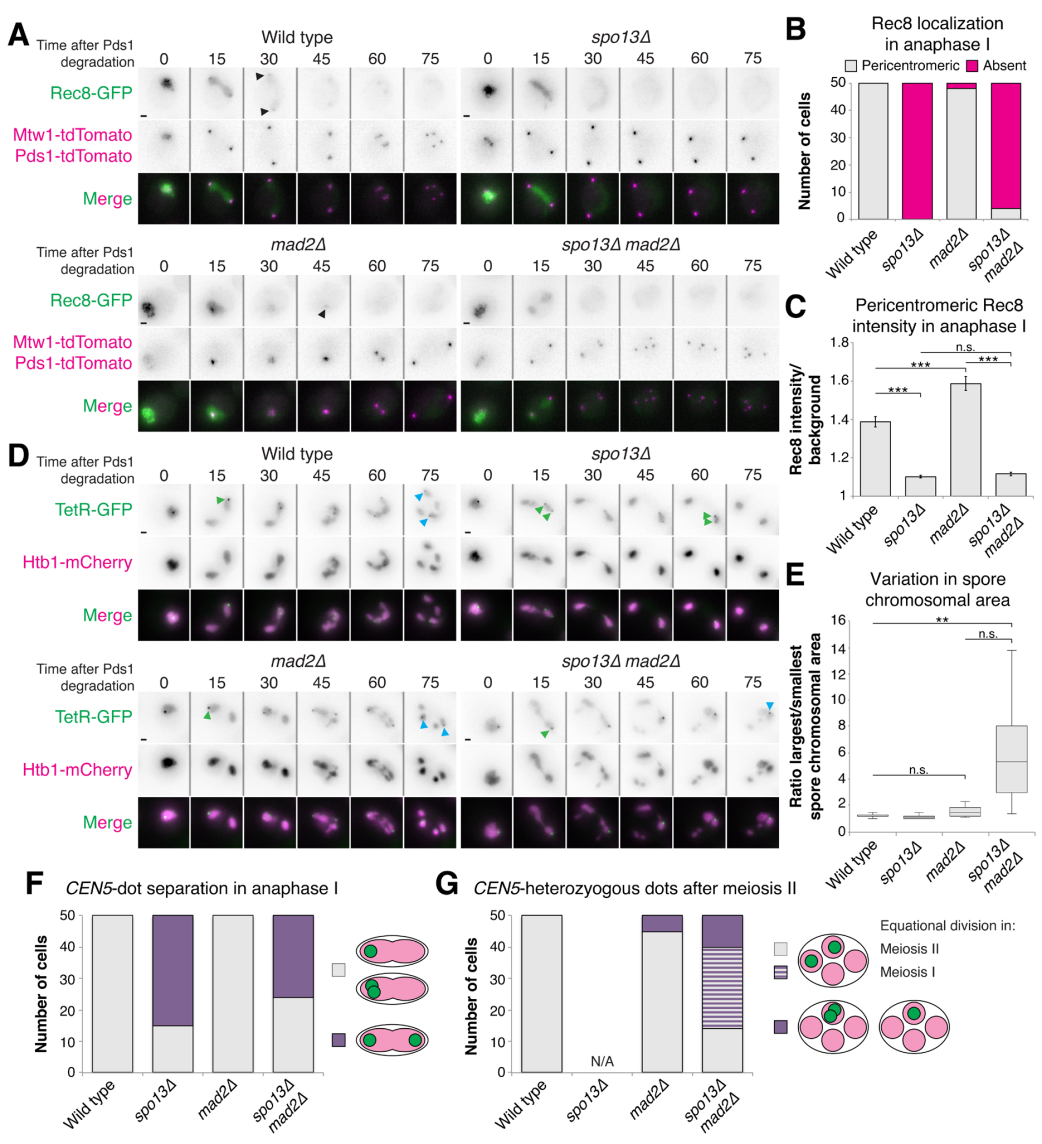
Deletion of *MAD2* restores the second nuclear division, but not accurate chromosome segregation to *spo13Δ* mutants. ( **A**– **C**) Representative images of Rec8-GFP, Mtw1-tdTomato and Pds1-tdTomato in live sporulating wild-type (AM13716), *spo13Δ* (AM24843), *mad2Δ* (AM24844) and *spo13Δ mad2Δ* (AM24845) cells. Scale bars represent 1 µm. Arrows indicate pericentromeric cohesin. ( **B**) The number of cells with pericentromeric Rec8-GFP in anaphase I is shown after scoring 50 cells from ( **A**). ( **C**) Rec8-GFP intensity was measured for 50 cells from ( **A**) as described for Figure 1C. ***p<0.001, n.s. = not significant (Welch two-sample t-test). For *spo13Δ mad2Δ* mutants, the analysis in ( **B**) and ( **C**) was performed exclusively for cells that performed two divisions (as judged by the presence of four Mtw1-tdTomato foci after meiosis II). ( **D**– **G**) Severe chromosome missegregation occurs in *spo13Δ mad2Δ* cells. ( **D**) Representative images of wild-type (AM24848), *spo13Δ* (AM24849), *mad2Δ* (AM25221) and *spo13Δ mad2Δ* (AM25222) cells carrying heterozygous TetR-GFP foci at *CEN5* and *HTB1-mCherry*. Green arrows indicate *CEN5*-GFP segregation outcomes after meiosis I, cyan arrows indicate *CEN5*-GFP segregation outcomes after meiosis II. ( **E**) Spores of *spo13Δ mad2Δ* vary greatly in the amount of nuclear DNA, as estimated by Htb1-mCherry area, thus indicating gross chromosome missegregation. The area occupied by Htb1-mCherry was measured in cells with four (wild type (n=45), *mad2Δ* (n=31) and *spo13Δ mad2Δ* (n=33)), or two ( *spo13Δ* (n=50)) nuclear masses after meiosis II and variation in chromosomal area estimated by obtaining the ratio of the largest and smallest nuclear mass for each cell. **p<0.01, n.s. = not significant (Welch two-sample t-test). ( **F**– **G**) *CEN5* missegregation in *spo13Δ mad2Δ* cells. Segregation of heterozygous *CEN5*-GFP foci was scored in 50 cells after the first ( **F**) and second ( **G**) nuclear division in the indicated strains. For *spo13Δ mad2Δ* mutants, the analysis in ( **F**) and ( **G**) was performed exclusively for cells that performed two divisions (as judged by the presence of four distinct Htb1-mCherry signals after meiosis II). Note that while a large proportion of *spo13Δ mad2Δ* cells end up with *CEN5*-GFP foci in two separate spores after meiosis II (similar to wild type), many of these cells have already segregated sister chromosomes in meiosis I (purple stripes), rather than meiosis II (gray).

We reasoned that the chromosome missegregation events seen in *spo13Δ* mutants might be related to the absence of the second nuclear division in these cells. Thus, restoring two divisions to *spo13Δ* cells by deletion of *MAD2* ( Shonn *et al*., 2002) would be expected to allow accurate chromosome segregation in the absence of Spo13. Our analysis of pericentromeric Rec8-GFP in anaphase I showed that, while pericentromeric cohesin in anaphase I is retained in wild-type and *mad2Δ* strains, it is lost to a similar degree in *spo13Δ* and *spo13Δ mad2Δ* mutants ( Figure 3A–C). Intriguingly, *mad2Δ* cells were frequently unable to separate kinetochores in anaphase I, despite successful cleavage of arm cohesin ( Figure 3A). While the reasons for this phenotype are unclear, we speculate that unattached kinetochores might persist into anaphase I when *MAD2* is deleted.

To analyse chromosome segregation in more detail, we followed cells carrying chromosomes labelled with Htb1-mCherry and heterozygous *CEN5*-GFP foci through meiosis ( Figure 3D). To assess global chromosome segregation, we assayed the chromosomal content of spores by measuring the area occupied by Htb1-mCherry after meiosis II and calculated the ratio of the largest and smallest chromosomal mass in each cell. In wild-type cells, this ratio is close to 1 in most cells ( Figure 3E) since all four nuclei are expected to be of similar size. In contrast, *spo13Δ mad2Δ* cells show a large variation in the chromosomal content of nuclei destined for spores ( Figure 3E), indicating gross chromosome missegregation. We additionally investigated the segregation of heterozygous *CEN5*-GFP foci in these cells ( Figure 3F, G). Similar to *spo13Δ* single mutants, a large proportion of *spo13Δ mad2Δ* double mutant cells split sister chromatids upon the first nuclear division ( Figure 3F). Furthermore, 20% of *spo13Δ mad2Δ* cells display *CEN5*-GFP dot(s) in only one out of four spores after meiosis II ( Figure 3G). This is largely caused by the absence of Spo13, since *mad2Δ* single mutants display a more modest missegregation phenotype ( Figure 3G). Therefore, *spo13Δ mad2Δ* cells fail to accurately segregate chromosomes during both the first and second nuclear divisions. We conclude that the lack of Spo13 causes loss of centromeric cohesion during meiosis I and severe chromosome missegregation even when the second nuclear division is restored.

### Sister chromatid cohesion is restored by preventing cohesin cleavage

**Figure 4.**
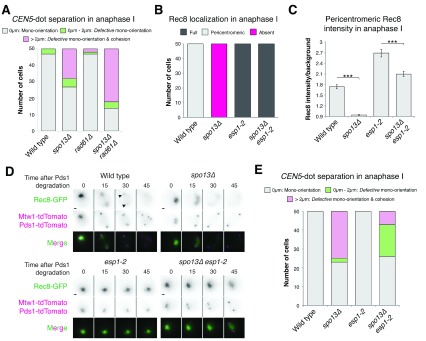
Cohesin protection in *spo13Δ* cells is rescued by inhibition of separase, but not by ablation of the prophase pathway. ( **A**) Deletion of *RAD61/WPL1* does not rescue sister chromatid cohesion in *spo13Δ* cells. Categorization of *CEN5-GFP* distances in wild-type (AM15190), *spo13Δ* (AM20146), *rad61Δ* (AM21068) and *spo13Δ rad61Δ* (AM21358) cells carrying *SPC42-tdTomato*, *PDS1-tdTomato* and heterozygous TetR-GFP dots at *CEN5* was carried out as described in Figure 2A. ( **B**– **D**) Separase activity is required for Rec8 removal in *spo13Δ* mutants. Wild-type (AM13716), *spo13Δ* (AM20033), *esp1-2* (AM20868) and *spo13Δ esp1-2* (AM21949) cells carrying *REC8-GFP*, *MTW1-tdTomato* and *PDS1-tdTomato* were resuspended in sporulation medium at 32°C and grown in flasks for 3h before transferring to a microfluidics plate and imaged at 32°C. ( **B**) The number of cells with the indicated patterns of Rec8-GFP localization in anaphase I was scored for 50 cells per strain. ( **C**) The intensity of pericentromeric Rec8-GFP for the indicated genotypes is shown. The mean of the two maximum intensity values on a straight line connecting both kinetochores in anaphase I (within the first two time points after Pds1-tdTomato degradation) was measured for 50 cells. Error bars represent standard error. ***p<0.001 (Welch two-sample t-test). ( **D**) Representative images are shown. Scale bars represent 1 µm. Arrows indicate pericentromeric cohesin. ( **E**) Inhibition of separase activity restores sister chromatid cohesion to *spo13Δ* mutants. Cohesion was assayed by categorization of *CEN5*-GFP ** distances as described in Figure 2A. Strains used were wild-type (AM15190), *spo13Δ* (AM20146), *esp1-2* (AM22498) and *spo13Δ esp1-2* (AM22499) cells carrying *SPC42-tdTomato*, *PDS1-tdTomato* and heterozygous TetR-GFP dots at *CEN5*.

A cleavage-independent, Rad61/Wpl1-dependent, cohesin removal pathway, similar to that which occurs in mammalian mitosis, operates during prophase I of budding yeast meiosis ( Challa *et al*., 2016; Challa *et al*., 2019; Yu & Koshland 2005). We considered the possibility that cells lacking Spo13 lose cohesion, not due to its cleavage, but as a result of ectopic Rad61 activity. However, deletion of *RAD61* did not restore cohesion to *spo13Δ* cells ( Figure 4A), indicating that a failure to counteract cleavage-independent cohesin removal is not solely responsible for the cohesion defect of cells lacking Spo13.

Next, we assessed whether cohesin cleavage is required for cohesion loss during anaphase I in *spo13Δ* cells. First, we inactivated Esp1 (separase), using the temperature-sensitive *esp1-2* mutant ( Buonomo *et al*., 2000) and followed Rec8-GFP by live cell imaging ( Figure 4B–D). As expected, cohesin remained on chromosomes even after anaphase I onset in both in *esp1-2* and *esp1-2 spo13Δ* cells and, consequently, sister chromatid segregation was largely prevented ( Figure 4E).

**Figure 5.**
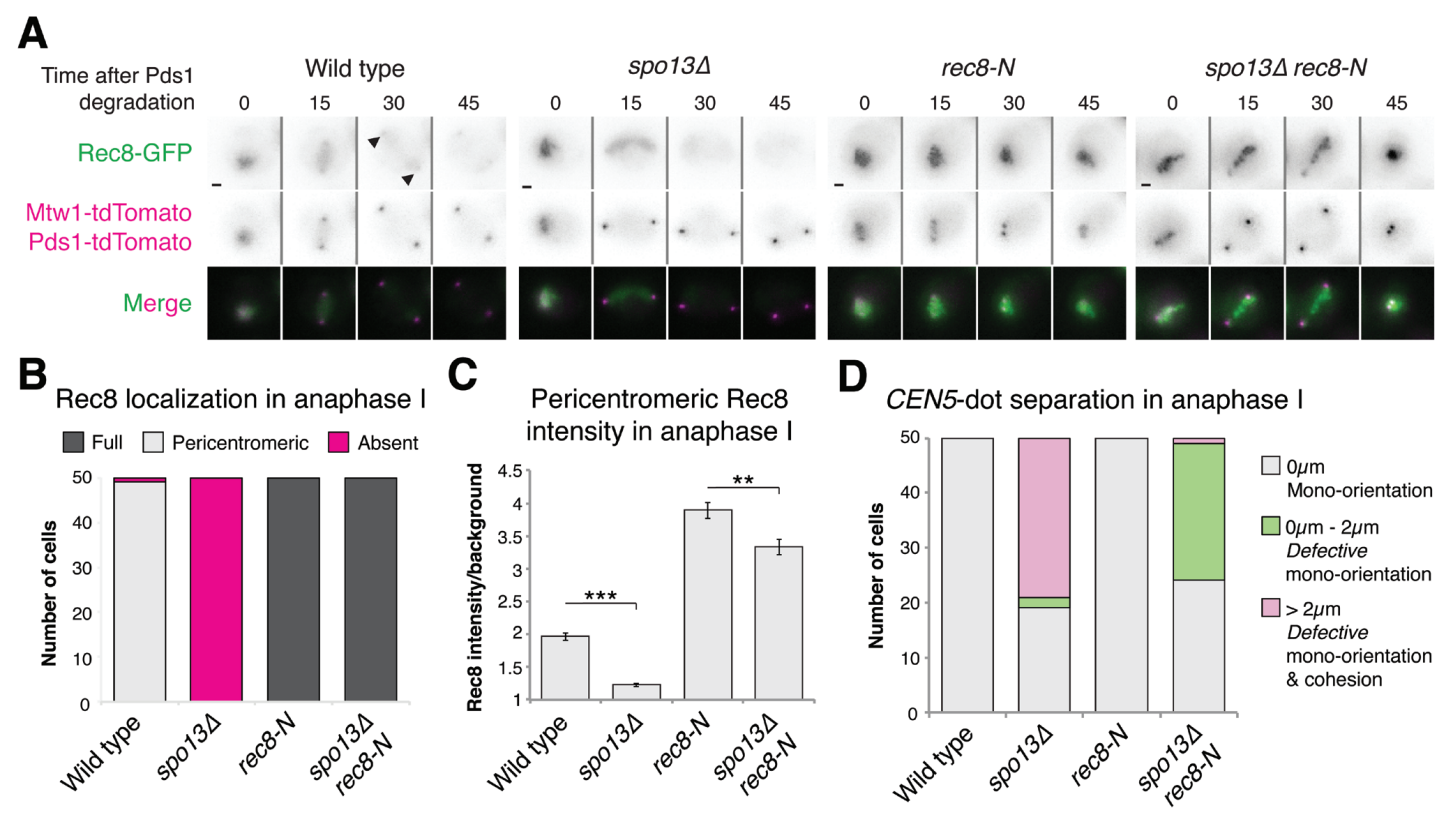
Cohesin cleavage is required for loss of sister chromatid cohesion in *spo13Δ* cells. ( **A**– **C**) Non-cleavable Rec8 blocks efficient removal of cohesin in *spo13Δ* cells. ( **A**) Representative images from movies of cells carrying Rec8-GFP, Mtw1-tdTomato, Pds1-tdTomato and with the indicated genotypes are shown. Scale bars represent 1 µm. Arrows indicate pericentromeric Rec8-GFP. ( **B**) Frequency of cells with the indicated pattern of Rec8-GFP localization is shown for the indicated genotypes. ( **C**) Rec8-GFP intensity was measured for the indicated genotypes as described in Figure 4C. Error bars represent standard error. **p<0.01, ***p<0.001 (Welch two-sample t-test). Strains used in ( **A**– **C**) were *REC8-GFP* (AM22190), *REC8-GFP spo13Δ* (AM22191), *rec8-N-GFP* (AM22192) and *rec8-N-GFP spo13Δ* (AM22193) cells carrying *MTW1-tdTomato* and *PDS1-tdTomato*. ( **D**) Non-cleavable Rec8 prevents sister chromatid segregation in *spo13Δ* mutants. Cohesion functionality was determined for the indicated genotypes by categorization of *CEN5-GFP* distances as described for Figure 2A. Strains were *REC8-3HA* (AM22346), *REC8-3HA spo13Δ* (AM22347), *rec8-N-3HA* (AM22348) and *rec8-N-3HA spo13Δ* (AM22349) and carried *SPC42-tdTomato*, *PDS1-tdTomato* and heterozygous TetR-GFP dots at *CEN5*.

Additionally, we prevented cohesin cleavage by mutating the separase cleavage site in Rec8 (Rec8-N) ( Buonomo *et al*., 2000). We followed GFP-tagged versions of this Rec8 variant through meiosis in wild- and *spo13Δ* cells ( Figure 5A). Similar to *esp1-2* mutants, *rec8-N* prevents cleavage of cohesin along the length of the chromosome in *spo13Δ* cells ( Figure 5B) and pericentromeric cohesin intensity is greatly increased ( Figure 5C). Furthermore, we find that Rec8-N prevented the segregation of sister chromatids in *spo13Δ* mutants ( Figure 5D). We conclude that cohesin cleavage is required for sister chromatid segregation in *spo13Δ* cells.

Interestingly, neither *esp1-2* ( Figure 4E) nor Rec8-N ( Figure 5D) prevented the splitting of sister centromeres in *spo13Δ* anaphase I, suggesting that pericentromeric cohesin may have been removed independently of cleavage in the absence of Spo13, allowing centromeres to come apart. However, cells lacking the mono-orientation protein Mam1 also split sister centromeres in anaphase I, despite intact pericentromeric cohesin protection ( Tóth *et al*., 2000). This suggests that the presence of uncleaved pericentromeric cohesin in anaphase I cannot prevent the sister centromere splitting resulting from defective mono-orientation in *spo13Δ* cells ( Katis *et al*., 2004b; Lee *et al*., 2004). Moreover, centromere breathing – the splitting of centromeres in response to spindle tension despite high concentrations of cohesin in the pericentromere – has been observed in pre-anaphase mitotic cells of multiple species ( Goshima & Yanagida, 2000; He *et al*., 2000; Nabeshima *et al*., 1998; Pearson *et al*., 2001; Shelby *et al*., 1996; Tanaka *et al*., 2000). Thus, the splitting of centromeres in anaphase I in *spo13Δ* in the absence of cohesin cleavage does not confirm cleavage-independent cohesin removal in the pericentromere.

### PP2A is functional in the absence of Spo13

**Figure 6.**
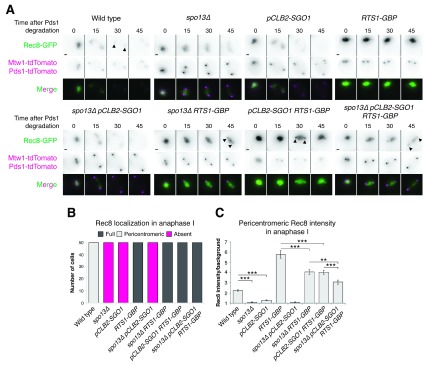
PP2A can prevent cohesin cleavage and sister chromatid segregation in *spo13Δ* cells. ( **A**– **C**) Cohesin is retained on chromosomes when PP2A ^Rts1^ is tethered to Rec8. ( **A**) Representative images of Rec8-GFP, Mtw1-dtTomato and Pds1-tdTomato in wild-type (AM13716), *spo13Δ* (AM20033), *pCLB2-SGO1* (AM21315), *RTS1-GBP* (AM21316), *spo13Δ pCLB2-SGO1* (AM21317), *spo13Δ RTS1-GBP* (AM21319), *pCLB2-SGO1 RTS1-GBP* (AM21318) and *spo13Δ pCLB2-SGO1 RTS1-GBP* (AM21320) cells undergoing meiosis. Scale bars represent 1 µm. Arrows indicate pericentromeric cohesin. ( **B**) The number of cells with pericentromeric cohesin in anaphase I was scored for 50 cells per strain. ( **C**) Rec8-GFP intensity in anaphase I was measured as described in Figure 2A. **p<0.01, ***p<0.001 (Welch two-sample t-test).

**Figure 7.**
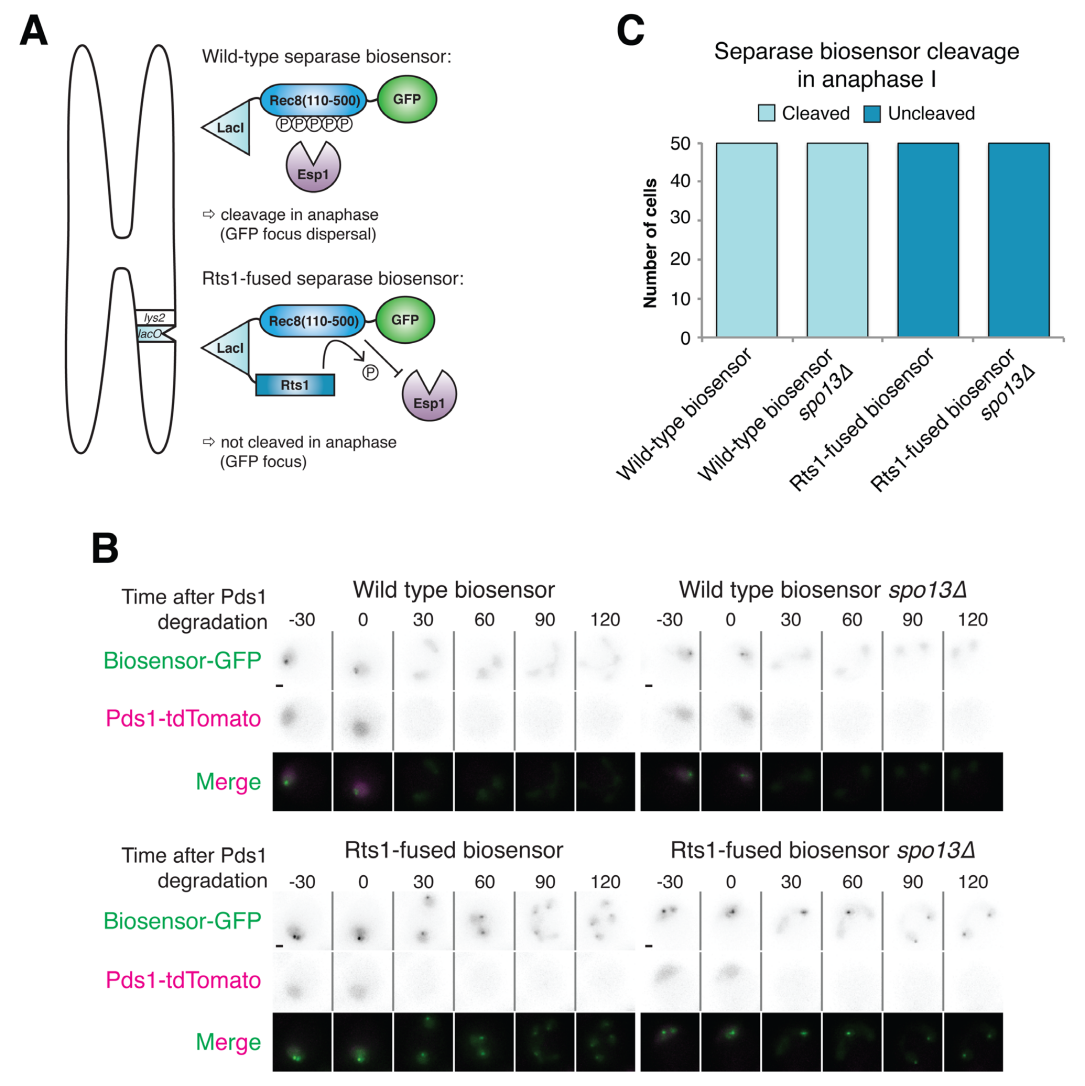
Fusion of Rts1 to a separase biosensor prevents its cleavage in both wild-type and *spo13Δ* cells. ( **A**) Schematic illustration of the separase biosensor and its Rts1 fusion. ( **B** and **C**) Wild-type (AM21557) and *spo13Δ* cells (AM21558) carrying a wild-type separase biosensor ( *pCUP1-GFP-REC8(110-500)-LacI*) or an Rts1 fused biosensor ( *pCUP1-GFP-REC8(110-500)-LacI-RTS1*; wild type: AM21559, *spo13Δ*: AM21800) as well as *lys2::lacOx256* and *PDS1-tdTomato* were sporulated in the presence of 100 nM CuSO _4_ for 2.5 h before imaging on a microfluidics plate. ( **B**) Representative images are shown. Scale bars represent 1 µm. ( **C**) Scoring of 50 cells per strain for the presence of GFP foci (uncleaved biosensor) or diffuse GFP signal (cleaved biosensor) within 30 min (two time points) of Pds1 degradation.

Rec8 cleavage during wild-type meiosis relies on its prior phosphorylation ( Brar *et al*., 2006; Katis *et al*., 2010) which is reversed in the pericentromere by PP2A. We considered the possibility that PP2A function may be impaired in *spo13Δ* cells, rendering it unable to dephosphorylate, and therefore protect, cohesin. We assessed whether tethering PP2A directly to cohesin could prevent Rec8 cleavage in the absence of Spo13. We fused GFP-binding protein (GBP), a nanobody specifically recognising GFP ( Rothbauer *et al*., 2006), to the PP2A regulatory subunit Rts1 to irreversibly tether PP2A to GFP-tagged Rec8. This was sufficient to prevent cohesin removal, both in *pCLB2-SGO1* and *spo13Δ* cells ( Figure 6A–C). To further confirm the full functionality of Rts1 in *spo13Δ* cells, we utilised a separase biosensor ( Yaakov *et al*., 2012) where a cleavable Rec8 moiety is fused to GFP and LacI, with the latter allowing targeting of the biosensor to a *lacO* array on chromosome arms ( Figure 7A). In wild-type and *spo13Δ* cells, this biosensor appears as a single GFP focus in meiosis I until separase is activated in anaphase I, causing biosensor cleavage and GFP focus dispersal ( Figure 7B, C). Tethering of Rts1 to the biosensor, however, prevents biosensor cleavage ( Figure 7B, C). Therefore, our results indicate that PP2A is functional and capable of dephosphorylating cohesin in *spo13Δ* mutants.

## Conclusions

The successful protection of pericentromeric cohesin is a key modification to the meiotic chromosome segregation machinery as it ensures the fidelity of chromosome segregation in meiosis II. Key players in regulating cohesin cleavage are known. The kinases Hrr25 and Cdc7 (and possibly Cdc5) phosphorylate cohesin along the length of the chromosome to promote its cleavage by separase ( Attner *et al*., 2013; Brar *et al*., 2006; Katis *et al*., 2010), while pericentromeric Sgo1 recruits the phosphatase PP2A to dephosphorylate Rec8 and thereby protect it ( Katis *et al*., 2004a; Kitajima *et al*., 2004; Lee *et al*., 2008 Marston *et al*., 2004; Riedel *et al*., 2006; Tang *et al*., 2006). However, the meiosis I-specific Spo13, is also required to retain pericentromeric cohesin in anaphase I ( Katis *et al*., 2004b; Lee *et al*., 2004; Shonn *et al*., 2002) but its function is much less well understood. Our study demonstrates that pericentromeric cohesin is prematurely removed in *spo13Δ* cells in a manner that requires cohesin cleavage and phosphorylation. Our recent work indicates that Spo13 achieves this by counteracting the activity of the cohesin kinases, Hrr25 and DDK ( Galander *et al*., 2019). Future work should focus on elucidating how Spo13 elicits its effects on kinase function, and how this might be linked to its functions in both sister kinetochore mono-orientation and meiotic cell cycle control.

## Methods

### Yeast strains and plasmids

All strains are SK1-derivatives and are listed in Table 1. Plasmids generated in this study are listed in Table 2. Gene deletions, promoter replacements and gene tags were introduced using PCR-based methods ( Gauss *et al*., 2005; Knop *et al*., 1999; Longtine *et al*., 1998; Moqtaderi & Struhl, 2008). *pCLB2-CDC20* ( Lee & Amon, 2003), *REC8-GFP*, *PDS1-tdTomato* ( Matos *et al*., 2008), *ndt80Δ* ( Vincenten *et al*., 2015), *SPC42-tdTomato* ( Fox *et al*., 2017), *REC8-3HA* ( Klein *et al*., 1999), *CEN5*-GFP dots, *mam1Δ::TRP1* ( Tóth *et al*., 2000) and *REC8-N* ( Buonomo *et al*., 2000) were described previously. Separase biosensor constructs ( Yaakov *et al*., 2012) were a kind gift from David Morgan (Departments of Physiology and Biochemistry and Biophysics, UCSF).

**Table 1.** *Saccharomyces cerevisiae* strains used in this study.

AM strain	Relevant genotype
4015	*ndt80Δ::LEU2/ndt80Δ::LEU2* *REC8-3HA::URA3/ REC8-3HA::URA3*
11633	*ndt80Δ::LEU2/ndt80Δ::LEU2*
13716	*REC8-GFP::URA3/ REC8-GFP::URA3* *PDS1-tdTomato::KITRP1/PDS1-tdTomato::KITRP1* *MTW1-tdTomato::NatMX6/MTW1-tdTomato::NatMX6*
15118	*SPC42-tdTomato::NatMX6/SPC42-tdTomato::NatMX6* *PDS1-tdTomato::KITRP1/PDS1-tdTomato::KITRP1"* *leu2::pURA3-TetR-GFP::LEU2/leu2::hisG* *CEN5::tetOx224::HIS3/CEN5* *spo13Δ::KanMX6/spo13Δ::KanMX6*
15119	*SPC42-tdTomato::NatMX6/SPC42-tdTomato::NatMX6* *PDS1-tdTomato::KITRP1/PDS1-tdTomato::KITRP1* *leu2::pURA3-TetR-GFP::LEU2/leu2::hisG* *CEN5::tetOx224::HIS3/CEN5* *mam1Δ::TRP1/mam1Δ::TRP1*
15120	*SPC42-tdTomato::NatMX6/SPC42-tdTomato::NatMX6* *PDS1-tdTomato::KITRP1/PDS1-tdTomato::KITRP1* *leu2::pURA3-TetR-GFP::LEU2/leu2::hisG* *CEN5::tetOx224::HIS3/CEN5* *spo13Δ::KanMX6/spo13Δ::KanMX6* *mam1Δ::TRP1/mam1Δ::TRP1*
15133	*REC8-GFP::URA3/ REC8-GFP::URA3* *PDS1-tdTomato::KITRP1/PDS1-tdTomato::KITRP1* *MTW1-tdTomato::NatMX6/MTW1-tdTomato::NatMX6* *spo13Δ::KanMX6/spo13Δ::KanMX6*
15134	*REC8-GFP::URA3/ REC8-GFP::URA3* *PDS1-tdTomato::KITRP1/PDS1-tdTomato::KITRP1* *MTW1-tdTomato::NatMX6/MTW1-tdTomato::NatMX6* *mam1Δ::TRP1/mam1Δ::TRP1*
15135	*REC8-GFP::URA3/ REC8-GFP::URA3* *PDS1-tdTomato::KITRP1/PDS1-tdTomato::KITRP1* *MTW1-tdTomato::NatMX6/MTW1-tdTomato::NatMX6* *spo13Δ::KanMX6/spo13Δ::KanMX6* *mam1Δ::TRP1/mam1Δ::TRP1*
15190	*SPC42-tdTomato::NatMX6/SPC42-tdTomato::NatMX6* *PDS1-tdTomato::KITRP1/PDS1-tdTomato::KITRP1* *leu2::pURA3-TetR-GFP::LEU2/leu2::hisG* *CEN5::tetOx224::HIS3/CEN5*
15342	*ndt80Δ::LEU2/ndt80Δ::LEU2* *REC8-3HA::URA3/REC8-3HA::URA3* *mam1Δ::TRP1/mam1Δ::TRP1*
15343	*ndt80Δ::LEU2/ndt80Δ::LEU2* *REC8-3HA::URA3/REC8-3HA::URA3* *spo13Δ::KanMX6/spo13Δ::KanMX6*
15344	*ndt80Δ::LEU2/ndt80Δ::LEU2* *REC8-3HA::URA3/REC8-3HA::URA3* *spo13Δ::KanMX6/spo13Δ::KanMX6* *mam1Δ::TRP1/mam1Δ::TRP1*
20033	*REC8-GFP::URA3/ REC8-GFP::URA3* *PDS1-tdTomato::KITRP1/PDS1-tdTomato::KITRP1* *MTW1-tdTomato::NatMX6/MTW1-tdTomato::NatMX6* *spo13Δ::HphMX6/spo13Δ::HphMX6*
20868	*REC8-GFP::URA3/ REC8-GFP::URA3* *PDS1-tdTomato::KITRP1/PDS1-tdTomato::KITRP1* *MTW1-tdTomato::NatMX6/MTW1-tdTomato::NatMX6* *esp1-2/esp1-2*
21068	*SPC42-tdTomato::NatMX6/SPC42-tdTomato::NatMX6* *PDS1-tdTomato::KITRP1/PDS1-tdTomato::KITRP1* *leu2::pURA3-TetR-GFP::LEU2/leu2::hisG* *CEN5::tetOx224::HIS3/CEN5* *rad61Δ::KanMX6/rad61Δ::KanMX6*
21315	*REC8-GFP::URA3/ REC8-GFP::URA3* *PDS1-tdTomato::KITRP1/PDS1-tdTomato::KITRP1* *MTW1-tdTomato::NatMX6/MTW1-tdTomato::NatMX6* *sgo1::KanMX6::pCLB2-SGO1/sgo1::KanMX6::pCLB2-SGO1*
21316	*REC8-GFP::URA3/ REC8-GFP::URA3* *PDS1-tdTomato::KITRP1/PDS1-tdTomato::KITRP1* *MTW1-tdTomato::NatMX6/MTW1-tdTomato::NatMX6* *RTS1-GBP::His3MX6/RTS1-GBP::His3MX6*
21317	*REC8-GFP::URA3/ REC8-GFP::URA3* *PDS1-tdTomato::KITRP1/PDS1-tdTomato::KITRP1* *MTW1-tdTomato::NatMX6/MTW1-tdTomato::NatMX6* *spo13Δ::HphMX6/spo13Δ::HphMX6* *sgo1::KanMX6::pCLB2-SGO1/sgo1::KanMX6::pCLB2-SGO1*
21318	*REC8-GFP::URA3/ REC8-GFP::URA3* *PDS1-tdTomato::KITRP1/PDS1-tdTomato::KITRP1* *MTW1-tdTomato::NatMX6/MTW1-tdTomato::NatMX6* *sgo1::KanMX6::pCLB2-SGO1/sgo1::KanMX6::pCLB2-SGO1* *RTS1-GBP::His3MX6/RTS1-GBP::His3MX6*
21319	*REC8-GFP::URA3/ REC8-GFP::URA3* *PDS1-tdTomato::KITRP1/PDS1-tdTomato::KITRP1* *MTW1-tdTomato::NatMX6/MTW1-tdTomato::NatMX6* *spo13Δ::HphMX6/spo13Δ::HphMX6* *RTS1-GBP::His3MX6/RTS1-GBP::His3MX6*
21320	*REC8-GFP::URA3/ REC8-GFP::URA3* *PDS1-tdTomato::KITRP1/PDS1-tdTomato::KITRP1* *MTW1-tdTomato::NatMX6/MTW1-tdTomato::NatMX6* *spo13Δ::HphMX6/spo13Δ::HphMX6* *sgo1::KanMX6::pCLB2-SGO1/sgo1::KanMX6::pCLB2-SGO1* *RTS1-GBP::His3MX6/RTS1-GBP::His3MX6*
21358	*SPC42-tdTomato::NatMX6/SPC42-tdTomato::NatMX6* *PDS1-tdTomato::KITRP1/PDS1-tdTomato::KITRP1* *leu2::pURA3-TetR-GFP::LEU2/leu2::hisG* *CEN5::tetOx224::HIS3/CEN5* *spo13Δ::HphMX6/spo13Δ::HphMX6* *rad61Δ::KanMX6/rad61Δ::KanMX6*
21557	*his3::pCUP1-GFP-REC8(110-500)-LacI::HIS3/his3::pCUP1-GFP-REC8(110-500)-LacI::HIS3* *lys2::LEU2::lacOx256/lys2::LEU2::lacOx256* *PDS1-tdTomato-KITRP1/PDS1-tdTomato-KITRP1*
21558	*his3::pCUP1-GFP-REC8(110-500)-LacI::HIS3/his3::pCUP1-GFP-REC8(110-500)-LacI::HIS3* > *lys2::LEU2::lacOx256/lys2::LEU2::lacOx256* *PDS1-tdTomato-KITRP1/PDS1-tdTomato-KITRP1* *spo13Δ::hphMX6/spo13Δ::hphMX6*
21559	*his3::pCUP1-GFP-REC8(110-500)-LacI-RTS1::HIS3/his3::pCUP1-GFP-REC8(110-500)-LacI-RTS1::HIS3* *lys2::LEU2::lacOx256/lys2::LEU2::lacOx256* *PDS1-tdTomato-KITRP1/PDS1-tdTomato-KITRP1*
21800	*his3::pCUP1-GFP-REC8(110-500)-LacI-RTS1::HIS3/his3::pCUP1-GFP-REC8(110-500)-LacI-RTS1::HIS3* *lys2::LEU2::lacOx256/lys2::LEU2::lacOx256* *PDS1-tdTomato-KITRP1/PDS1-tdTomato-KITRP1* *spo13Δ::hphMX6/spo13Δ::hphMX6*
21949	*REC8-GFP::URA3/ REC8-GFP::URA3* *PDS1-tdTomato::KITRP1/PDS1-tdTomato::KITRP1* *MTW1-tdTomato::NatMX6/MTW1-tdTomato::NatMX6* *spo13Δ::HphMX6/spo13Δ::HphMX6* *esp1-2/esp1-2*
22190	*rec8::REC8-GFP::LEU2::KanMX4/rec8::REC8-GFP::LEU2::KanMX4* *MTW1-tdTomato::NatMX6/MTW1-tdTomato::NatMX6* *PDS1-tdTomato::KITRP1/PDS1-tdTomato::KITRP1*
22191	*rec8::REC8-GFP::LEU2::KanMX4/rec8::REC8-GFP::LEU2::KanMX4* *MTW1-tdTomato::NatMX6/MTW1-tdTomato::NatMX6* *PDS1-tdTomato::KITRP1/PDS1-tdTomato::KITRP1* *spo13Δ::HphMX6/spo13Δ::HphMX6*
22192	*rec8::rec8-N-GFP::LEU2::KanMX4/rec8::rec8-N-GFP::LEU2::KanMX4* *MTW1-tdTomato::NatMX6/MTW1-tdTomato::NatMX6* *PDS1-tdTomato::KITRP1/PDS1-tdTomato::KITRP1*
22193	*rec8::rec8-N-GFP::LEU2::KanMX4/rec8::rec8-N-GFP::LEU2::KanMX4* *MTW1-tdTomato::NatMX6/MTW1-tdTomato::NatMX6* *PDS1-tdTomato::KITRP1/PDS1-tdTomato::KITRP1* *spo13Δ::HphMX6/spo13Δ::HphMX6*
22346	*SPC42-tdTomato::NatMX6/SPC42-tdTomato::NatMX6* *PDS1-tdTomato::KITRP1/PDS1-tdTomato::KITRP1* *leu2::pURA3-TetR-GFP::LEU2/leu2::hisG* *CEN5::tetOx224::HIS3/CEN5* *rec8::REC8-3HA::LEU2::KanMX4/rec8::REC8-3HA::LEU2::KanMX4*
22347	*SPC42-tdTomato::NatMX6/SPC42-tdTomato::NatMX6* *PDS1-tdTomato::KITRP1/PDS1-tdTomato::KITRP1* *leu2::pURA3-TetR-GFP::LEU2/leu2::hisG* *CEN5::tetOx224::HIS3/CEN5* *rec8::REC8-3HA::LEU2::KanMX4/rec8::REC8-3HA::LEU2::KanMX4* *spo13Δ::HphMX6/spo13Δ::HphMX6*
22348	*SPC42-tdTomato::NatMX6/SPC42-tdTomato::NatMX6* *PDS1-tdTomato::KITRP1/PDS1-tdTomato::KITRP1* *leu2::pURA3-TetR-GFP::LEU2/leu2::hisG* *CEN5::tetOx224::HIS3/CEN5* *rec8::rec8-N-3HA::LEU2::KanMX4/rec8::rec8-N-3HA::LEU2::KanMX4*
22349	*SPC42-tdTomato::NatMX6/SPC42-tdTomato::NatMX6* *PDS1-tdTomato::KITRP1/PDS1-tdTomato::KITRP1* *leu2::pURA3-TetR-GFP::LEU2/leu2::hisG* *CEN5::tetOx224::HIS3/CEN5* *rec8::rec8-N-3HA::LEU2::KanMX4/rec8::rec8-N-3HA::LEU2::KanMX4* *spo13Δ::HphMX6/spo13Δ::HphMX6*
22498	*SPC42-tdTomato::NatMX6/SPC42-tdTomato::NatMX6* *PDS1-tdTomato::KITRP1/PDS1-tdTomato::KITRP1* *leu2::pURA3-TetR-GFP::LEU2/leu2::hisG* *CEN5::tetOx224::HIS3/CEN5* *esp1-2/esp1-2*
22499	*SPC42-tdTomato::NatMX6/SPC42-tdTomato::NatMX6* *PDS1-tdTomato::KITRP1/PDS1-tdTomato::KITRP1* *leu2::pURA3-TetR-GFP::LEU2/leu2::hisG* *CEN5::tetOx224::HIS3/CEN5* *spo13Δ::HphMX6/spo13Δ::HphMX6* *esp1-2/esp1-2*
24843	*REC8-GFP::URA3/ REC8-GFP::URA3* *PDS1-tdTomato::KITRP1/PDS1-tdTomato::KITRP1* *MTW1-tdTomato::NatMX6/MTW1-tdTomato::NatMX6* *spo13Δ::hisG/spo13Δ::hisG*
24844	*REC8-GFP::URA3/ REC8-GFP::URA3* *PDS1-tdTomato::KITRP1/PDS1-tdTomato::KITRP1* *MTW1-tdTomato::NatMX6/MTW1-tdTomato::NatMX6* *mad2Δ::KanMX6/mad2Δ::KanMX6*
24845	*REC8-GFP::URA3/ REC8-GFP::URA3* *PDS1-tdTomato::KITRP1/PDS1-tdTomato::KITRP1* *MTW1-tdTomato::NatMX6/MTW1-tdTomato::NatMX6* *spo13Δ::hisG/spo13Δ::hisG* *mad2Δ::KanMX6/mad2Δ::KanMX6*
24848	*leu2::pURA3-TetR-GFP::LEU2/leu2::hisG* *CEN5::tetOx224::HIS3/CEN5* *HTB1-mCherry::His3MX6/HTB1-mCherry::His3MX6*
24849	*leu2::pURA3-TetR-GFP::LEU2/leu2::hisG* *CEN5::tetOx224::HIS3/CEN5* *HTB1-mCherry::His3MX6/HTB1-mCherry::His3MX6* *spo13Δ::KanMX6/spo13Δ::KanMX6*
25221	*leu2::pURA3-TetR-GFP::LEU2/leu2::hisG* *CEN5::tetOx224::HIS3/CEN5* *HTB1-mCherry::His3MX6/HTB1-mCherry::His3MX6* *mad2Δ::HphMX6/mad2Δ::HphMX6*
25222	*leu2::pURA3-TetR-GFP::LEU2/leu2::hisG* *CEN5::tetOx224::HIS3/CEN5* *HTB1-mCherry::His3MX6/HTB1-mCherry::His3MX6* *spo13Δ::KanMX6/spo13Δ::KanMX6* *mad2Δ::HphMX6/mad2Δ::HphMX6*

**Table 2.** Plasmids generated in this study.

Plasmid	Description	Purpose and notes
AMp1317	YIplac128-REC8-GFP	*LEU2* integration plasmid carrying *REC8-GFP*.
AMp1368	YIplac128-rec8-N-GFP	*LEU2* integration plasmid carrying *rec8-N-GFP*.

### Growth conditions

Cells were prepared for sporulation as described by Vincenten *et al*. (2015).

### Chromatin immunoprecipitation

ChIP-qPCR was performed as previously described ( Vincenten *et al*., 2015), using mouse anti-Ha (12CA5, Roche). All parameters and equipment are identical to those described previously, including qPCR mixes and thermocycling conditions. Primers for qPCR analysis are listed in Table 3.

**Table 3.** qPCR primers used in this study. For distances from centromeres, “-“ indicates the location is upstream of the centromere, whereas “+” indicates the location is downstream of the centromere.

Chr.	Location	Distance from centromere	Primer pair	Sequence
III	Centromere	+0.25kb	1279	TGTTGATGGGTTTACAATTT
			1280	CTTTCAATGATTGCTCTAAATC
IV	Arm	-95kb	782	AGATGAAACTCAGGCTACCA
			783	TGCAACATCGTTAGTTCTTG
IV	Centromere	+0.15kb	794	CCGAGGCTTTCATAGCTTA
			795	ACCGGAAGGAAGAATAAGAA

### Live cell imaging

Live cell imaging was performed on a DeltaVision Elite system (Applied Precision) connected to an inverted Olympus IX-71 microscope with a 100x UPlanSApo NA 1.4 oil lens. Images were taken using a Photometrics Cascade II EMCCD camera. The Deltavision system was controlled using SoftWoRx software, version 5.5. Live-cell imaging for Figure 3 was performed on a Zeiss Axio Observer Z1 (Zeiss UK, Cambridge) equipped with a Hamamatsu Flash 4 sCMOS camera, Prior motorised stage and Zen 2.3 acquisition software.

Cells were imaged at 30˚C (unless stated) on an ONIX microfluidic perfusion platform by CellASIC. Cells were pre-grown in culture flasks for ~3 h before transfer to microfluidics plates. Imaging began about 30 min later with images being acquired every 15 min for 12-15 h. Seven z-stacks were acquired with 0.85µm spacing. Image panels were assembled using Image-Pro Premier 3D, version 9.1 (Media Cybernetics). Images were analysed using ImageJ 1.48v (National Institutes of Health). Final image assembly was carried out using Adobe Photoshop CS5.1 and Adobe Illustrator CS5.1. Rec8-GFP intensities were measured using the DV_DotCounter custom plugin for ImageJ ( Kelly, 2019a). The plugin applied a Z projection to each colour channel and allowed the user to select a cell of interest. Kinetochores in the red channel were identified by Yen Auto Threshold ( Yen *et al*., 1995) and their XY central coordinates, mean intensity and area recorded. The coordinates were then used to measure mean intensity in the corresponding location in the green channel, equivalent to pericentromeric Rec8-GFP. In experiments where pericentromeric cohesin was likely to be found in between kinetochores (which is thought to occur in cells that bi-orient in meiosis I but retain cohesin), the XY coordinates in the red channel were used to generate a line profile between the 2 kinetochores in both colour channels over exactly the same pixels. The two brightest peaks in the line profile of the green channel were calculated to give the maximum intensity value for each. Rec8-GFP intensity was measured in this manner for Figure 4C and Figure 5C. The plugin used was the custom YeastLineProfiler for ImageJ ( Kelly, 2019b). Chromosomal area in Figure 3E was measured using a custom ImageJ plugin ( Kelly, 2019c) that identifies the regions of bright fluorescence in the red channel using Yen Auto Threshold and subsequently measures the area of these regions of interest.

An earlier version of this article can be found on bioRxiv (DOI: https://doi.org/10.1101/488312)

## Data availability

Raw data for scoring imaging experiments and ChIP-qPCR, arranged by figure, is available from OSF. DOI: https://doi.org/10.17605/OSF.IO/VBKU9 ( Marston, 2019).

Data are available under the terms of the Creative Commons Zero “No rights reserved” data waiver (CC0 1.0 Public domain dedication).

The file size of the raw microscopy movies precludes uploading them to OSF, but are available upon request from adele.marston@ed.ac.uk.

## Software availability

Source code for DV_DotCounter is available from: https://github.com/dkelly604/DV_DotCounter.

Archived source code at time of publication: https://doi.org/10.5281/zenodo.2553081 ( Kelly, 2019a).

Source code for YeastLineProfiler is available from: https://github.com/dkelly604/YeastLineProfiler.

Archived source code at time of publication: http://doi.org/10.5281/zenodo.2560099 ( Kelly, 2019b).

Source code for Size_and_Area is available from: https://github.com/dkelly604/Size_and_Area.

Archived source code at time of publication: http://doi.org/10.5281/zenodo.3358842 ( Kelly, 2019c).

License: MIT License.
