# Supplementary material for: Spo13 prevents premature cohesin cleavage during meiosis
Source: Wellcome Open Res. 2019 Sep 2;4:29. Originally published 2019 Feb 13. [Version 2] doi: 10.12688/wellcomeopenres.15066.2 (PMC6426077; doi:10.12688/wellcomeopenres.15066.2)
Supplement: Peer review history

# Peer review: Reviewer response for version 2

**DOI:** 10.21956/wellcomeopenres.16829.r36375

**Published:** 2019-09-10

**Authors:** Andreas Hochwagen

**Affiliations:** 1 Department of Biology, New York University, New York City, NY, USA

The revised manuscript adequately addresses all points raised in my review of the original manuscript.

I confirm that I have read this submission and believe that I have an appropriate level of expertise to confirm that it is of an acceptable scientific standard.

# Peer review: Reviewer response for version 1

**DOI:** 10.21956/wellcomeopenres.16436.r34862

**Published:** 2019-03-19

**Authors:** Enrique Martinez-Perez

**Affiliations:** 1 MRC London Institute of Medical Sciences, Imperial College London, London, UK

Accurate formation of haploid gametes during meiosis requires the step-wise removal of cohesin during the consecutive meiotic divisions. Two different pathways contribute to this process, one mediated by Wapl, which removes cohesin before the onset of the first meiotic division, and a second dependent on the protease separase, which cleaves the kleisin subunit (Rec8) of cohesin at the onset of the meiotic divisions. Phosphorylation of Rec8 by multiple kinases promotes cohesin release by Wapl and cleavage by separase, therefore the pool of cohesin bound to centromeric regions must be protected from phosphorylation during the first meiotic division to prevent premature loss of cohesion. Galander et al. use in vivo imaging to investigate cohesin protection in budding yeast, focusing on Spo13, which role in this process remains poorly understood.

 The authors show convincingly that spo13 mutants display premature loss of Rec8 and sister chromatid cohesion during the first meiotic division, that this premature loss of cohesion requires separase but not Wapl, and that expression of a separase-resistant Rec8 rescues cohesin loss in spo13 mutants. These results demonstrate an important role for Spo13 in preventing separase-dependent Rec8 removal during meiosis I.

 Specific comments:

 Figure 4 shows that expression of Rec8-N (separase resistant) prevents loss of Rec8 from pericentromeric regions in spo13 mutants, but despite this, sister centromeres still show substantial separation in ~50% of the cells. How can sister centromeres achieve this level of separation despite extensive Rec8 binding?

 The introduction doesn’t mention the Challa et al 2019 ^1^ paper describing the role of Wapl in promoting Rec8 removal before anaphase I. Since distinguishing the contribution of the Wapl and separase pathways to the cohesion defects of spo13 mutants is a key aspect of the manuscript, mentioning the Challa et al 2019 in the introduction will help the reader.

 Non-yeast experts will benefit from a more detailed description of some of the markers used in the study, such as Mtw1.

I confirm that I have read this submission and believe that I have an appropriate level of expertise to confirm that it is of an acceptable scientific standard.

## References

[1] : Meiosis-specific prophase-like pathway controls cleavage-independent release of cohesin by Wapl phosphorylation. *PLoS Genet*.15(1) : 10.1371/journal.pgen.1007851 e1007851 10.1371/journal.pgen.1007851 30605471PMC6317811

# Response

**Published:** 2019-08-05

**Authors:** Adele Marston

**Affiliations:** University of Edinburgh, UK

*Figure 4 shows that expression of Rec8-N (separase resistant) prevents loss of Rec8 from pericentromeric regions in spo13 mutants, but despite this, sister centromeres still show substantial separation in ~50% of the cells. How can sister centromeres achieve this level of separation despite extensive Rec8 binding?*

**

 Please refer to our comment on the final point made by reviewer 2. Sister kinetochores are bioriented.

*The introduction doesn’t mention the Challa et al 2019 ^1^ paper describing the role of Wapl in promoting Rec8 removal before anaphase I. Since distinguishing the contribution of the Wapl and separase pathways to the cohesion defects of spo13 mutants is a key aspect of the manuscript, mentioning the Challa et al 2019 in the introduction will help the reader.*

 We agree and are sorry for this omission. We have now done this.

*Non-yeast experts will benefit from a more detailed description of some of the markers used in the study, such as Mtw1.*

 We have clarified the function of Mtw1 in our updated manuscript and also provided a better explanation of the *mam1* *Δ* phenotype.

# Peer review: Reviewer response for version 1

**DOI:** 10.21956/wellcomeopenres.16436.r34866

**Published:** 2019-03-14

**Authors:** Dean S. Dawson

**Affiliations:** 1 Program in Cell Cycle and Cancer Biology, Oklahoma Medical Research Foundation, Oklahoma City, OK, USA

Galander and colleagues have revisited the role of Spo13 in protecting or regulating sister chromatid cohesion in meiosis I. Previous work has shown that Spo13 affects the disposition of cohesin at centromeres but the molecular basis for the modulation of centromeric cohesion by Spo13 has remained unclear. Recent work has shown that there are two pathways for cohesin removal in meiosis in budding yeast. Here the authors test which pathway is impacted by Spo13. The experiments have moved the field forward by using live cell imaging methods to address this question. The results demonstrate that Spo13, at least in part, protects Rec8 at the centromeres from cleavage by separase.

**Introduction**

 Para. 3, line 7. Might be good to adjust the sentence saying Rec8 cleavage is dependent on Cdc5. The next sentence indicates the lack of clarity on this point. 

 Paragaph 4: It would be worth mentioning the two-step removal of cohesins in budding yeast meiosis here (Yu and Koshland, 2005 ^1^; Challa et al., 2019 ^2^), along with referencing the mammalian and fly two-step processes.

**Results and Discussion**

 Page 4, second column, 4 lines from bottom: “withbi”

 Fig. 3 E/Fig. 4 D – spo13 delete allows meiosis I sister centromere separation in esp1 and REC8-N mutants.  This shows centromeres are more able to separate in spo13 mutants even without Rec8 cleavage. There are multiple possible explanations for these results. Is it because sister centromeres are more easily bi-oriented in spo13 mutants? Alternatively, could it be that Spo13 also promotes sister centromere cohesion also protects pericentromeric cohesion through a pathway that doesn’t involve cleavage? The manuscript would benefit from brief comments from the authors on the implications of these observations.

I confirm that I have read this submission and believe that I have an appropriate level of expertise to confirm that it is of an acceptable scientific standard.

## References

[1] : Chromosome morphogenesis: condensin-dependent cohesin removal during meiosis. *Cell*.2005;123(3) : 10.1016/j.cell.2005.09.014 397-407 10.1016/j.cell.2005.09.014 16269332

[2] : Meiosis-specific prophase-like pathway controls cleavage-independent release of cohesin by Wapl phosphorylation. *PLoS Genet*.15(1) : 10.1371/journal.pgen.1007851 e1007851 10.1371/journal.pgen.1007851 30605471PMC6317811

# Response

**Published:** 2019-08-05

**Authors:** Adele Marston

**Affiliations:** University of Edinburgh, UK

*Para. 3, line 7. Might be good to adjust the sentence saying Rec8 cleavage is dependent on Cdc5. The next sentence indicates the lack of clarity on this poin*t. 

 Thanks for the suggestion, we have done this.

*Paragaph 4: It would be worth mentioning the two-step removal of cohesins in budding yeast meiosis here (Yu and Koshland, 2005 ^1^; Challa et al., 2019 ^2^), along with referencing the mammalian and fly two-step processes.*

**

 Thanks for pointing out this omission, we have added the suggested text and references.

***Results and Discussion***

**

*Page 4, second column, 4 lines from bottom: “withbi”*

 This has been corrected 

*Fig. 3 E/Fig. 4 D – spo13 delete allows meiosis I sister centromere separation in esp1 and REC8-N mutants.  This shows centromeres are more able to separate in spo13 mutants even without Rec8 cleavage. There are multiple possible explanations for these results. Is it because sister centromeres are more easily bi-oriented in spo13 mutants? Alternatively, could it be that Spo13 also promotes sister centromere cohesion also protects pericentromeric cohesion through a pathway that doesn’t involve cleavage? The manuscript would benefit from brief comments*

*from the authors on the implications of these observations.*

 We believe that this is due to the fact that sister centromeres are bioriented in *spo13* mutants. We have added a few sentences explaining these observations and the previous evidence that shows that defects in monoorientation result in sister centromere splitting even without loss of cohesion.

# Peer review: Reviewer response for version 1

**DOI:** 10.21956/wellcomeopenres.16436.r34863

**Published:** 2019-02-27

**Authors:** Andreas Hochwagen

**Affiliations:** 1 Department of Biology, New York University, New York City, NY, USA

This manuscript by Galander and colleagues investigates the function of the Spo13, a central but poorly understood regulator of meiotic chromosome segregation, using live-cell imaging in *S. cerevisiae*. Previous work had shown that Spo13 weakens the protection of centromeric cohesin during meiosis I, but the extent of this weakening had been questioned. The authors show an essentially complete loss of centromeric cohesin by fluorescence microscopy and support this data by monitoring the segregation of sister centromeres. In addition, they propose that this weakening occurs through increased separase activity because of reduced cohesin phosphorylation in the pericentromeric regions. The latter conclusion is complicated by the fact that the phosphorylation experiments do not distinguish between cohesion at centromeres and along chromosome arms.

 I suggest the authors address the following points:

1. The data shown in panel 1C is central to the presented conclusions. As this bar graph relies on standard error, please provide statistical analysis including multiple hypothesis testing for this graph. 
2. I am confused why there are cells that did not lose cohesion in the mam1 spo13 double mutants (panel 2B). Does this data not indicate that there may be some cohesion remaining in the absence of Spo13? 
3. The Rts1-GBP construct will lead to ectopic protection also along chromosome arms. This increased signal is expected lead to an elevated Rec8 fluorescence intensity in the pericentromeric regions given the low spatial resolution of this assay. I think a ChIP experiment comparing Rec8 at arm and pericentromeric sites would be important to exclude the possibility that there is Rts1-independent Rec8 removal in the spo13 mutant. This issue is particularly relevant given a recent paper by Mehta et al ^1^ (Curr Genet) that came to the conclusion that Spo13 acts independently of Rts1.

 Typo:

 Page 4, second paragraph: withbi-oriented

I confirm that I have read this submission and believe that I have an appropriate level of expertise to confirm that it is of an acceptable scientific standard, however I have significant reservations, as outlined above.

## References

[1] : An interplay between Shugoshin and Spo13 for centromeric cohesin protection and sister kinetochore mono-orientation during meiosis I in Saccharomyces cerevisiae. *Curr Genet*.2018;64(5) : 10.1007/s00294-018-0832-x 1141-1152 10.1007/s00294-018-0832-x 29644457

# Response

**Published:** 2019-08-05

**Authors:** Adele Marston

**Affiliations:** University of Edinburgh, UK

1. We performed statistical analysis using Welch’s two sample t-test, which suggests that the observed differences are highly significant.

 2. While we agree that this is a possibility, we believe that there are alternative explanations for this observation. Firstly, it could be that, for unknown reasons, the deletion of *MAM1* affects successful segregation of chromosomes in anaphase I in *spo13* *Δ* cells (which otherwise retain some monopolin at kinetochores (Lee et al. (2004) Curr Biol 14, 2168-2182). Indeed, in Figure 2B, as well as additional figures in this manuscript (new Figures 4E and 5D), the fraction of otherwise wild-type *spo13* *Δ* cells that fail to segregate sister chromatids is minimal (2%). Secondly, the cells that appear to retain cohesin may be in the process of losing cohesion but our imaging could have captured them immediately after Pds1 degradation, so that they have not yet had a chance to segregate chromosomes before we scored them. To further test whether cohesion is retained at centromeres in the absence of Spo13, we analysed cells that also lack Mad2 and which have previously been found to undergo two divisions. This data, shown in a new Figure 3, confirms our previous conclusions that Spo13 is required for cohesion protection.

 3. While we agree that Rts1-independent Rec8 removal in the *spo13Δ* mutant is an interesting possibility, we decided not to do this experiment because our opinion is that it would be extremely difficult to interpret due to the difficulty of obtaining comparable cell cycle stages in the different mutants. The experiment would require analysis of cells in anaphase I (or at least past metaphase I) and, due to the metaphase I delay observed in *spo13Δ* cells even when synchronised using the *GAL-NDT80* block release system (Carlile and Amon, 2008), it would be difficult to obtain populations in which similar numbers of cells of each mutant are in this stage. Furthermore, it would be difficult to assess the cell cycle stage, since RTS1-GBP cells would presumably not exhibit standard anaphase spindles due to the failure to segregate chromosomes in anaphase I (compare distances of kinetochores in our imaging at the 15 minute time point in Figure 6A of the updated version of the manuscript). Additionally, *spo13Δ* mutants prematurely exit meiosis after the first division, precluding cell cycle analysis by immublot against an anaphase I marker. However, our main conclusion from these experiments is that PP2A-Rts1 is functional in the absence of Spo13 and we believe are data supports this interpretation.

 We also respectfully disagree with the conclusion in Mehta et al. based on the available data. Their conclusion that Spo13 acts independently of Rts1 was derived from data presented in Figure 3 of their paper where they showed that *CEN5-GFP *foci segregate to opposite poles during anaphase I in more *spo13* *Δ rts1Δ *than *spo13 *cells. However, these genotypes are not complete: the methods section and strain table reveal that all *spo13* *Δ *cells (but none of the other mutations) also carry *mad2* *Δ *delete to allow the cells to go through two meiotic divisions. Therefore, this experiment is not properly controlled, especially because *mad2 *cells themselves are compromised in chromosome segregation (Figure 3 in our current manuscript).

 In conclusion, while Rts1-independent Rec8 removal by Spo13 remains a possibility, there is currently no evidence for it.

 The typo on Page 4 has been corrected.
